# Effects of Secreted Mast Cell Mediators on Retinal Pigment Epithelial Cells: Focus on Mast Cell Tryptase

**DOI:** 10.1155/2017/3124753

**Published:** 2017-06-29

**Authors:** Rei Arai, Ayumi Usui-Ouchi, Yosuke Ito, Keitaro Mashimo, Akira Murakami, Nobuyuki Ebihara

**Affiliations:** ^1^Department of Ophthalmology, Juntendo University Urayasu Hospital, Chiba, Japan; ^2^Department of Ophthalmology, Juntendo University School of Medicine, Tokyo, Japan

## Abstract

Numerous mast cells are present in the choroid, but the effects of mast cell mediators on retinal pigment epithelial (RPE) cells are not well understood. We investigated the influence of mast cell mediators on RPE cells in vitro, focusing on tryptase. Expression of receptors was examined by the reverse transcription polymerase chain reaction. We also assessed production of interleukin 8 and vascular endothelial growth factor (VEGF) after RPE cells were stimulated with mast cell mediators by using an antibody array and enzyme-linked immunosorbent assay. Furthermore, we investigated the influence of tryptase on RPE cell migration and integrity by the scratch assay and the transepithelial resistance. RPE cells expressed protease-activated receptor 2 (PAR2), histamine receptor 1, tumor necrosis factor-*α* (TNF-*α*) receptor 1, and CCR 1, 3, 4, 8, and 11. Tryptase, PAR2 agonists, histamine, and TNF-*α* all enhanced interleukin 8 production by RPE cells, while only tryptase enhanced VEGF production. Tryptase also enhanced expression of phosphorylated extracellular signal-regulated kinases 1/2, resulting in increased migration of RPE cells. However, tryptase did not alter epithelial integrity or the expression of zonula occludens-1 and junctional adhesion molecule-A by RPE cells. Mast cell mediators, especially tryptase, may influence RPE cell inflammation.

## 1. Introduction

Mast cells are abundant in the choroid, whereas only a few of these cells are found in the anterior uvea. Choroidal mast cells are frequently located near the blood vessels in the inner vascular layer of the choroid [1–3], while these cells decrease in the outer choroidal layer and there are only a few mast cells in the suprachoroid [1, 4]. There are two distinct mast cell subtypes in humans that are distinguished by the neutral proteases in their granules, with the T subtype only having tryptase in its granules, while granules of the TC subtype contain both tryptase and chymase. It was reported that most choroidal mast cells belong to the TC subtype with granules containing both chymase and tryptase, and this was confirmed by investigation of choroidal mast cell suspensions [1–3, 5]. Miller et al. demonstrated that human choroidal mast cells respond to various immunological and nonimmunological stimuli [5]. For example, degranulation occurs after exposure to antihuman IgE antibody, compound 48/80, morphine, and calcium ionophore A23187, resulting in the release of various mediators. Therefore, numerous mast cells capable of releasing various mediators reside in the inner vascular layer of the choroid. Although mast cells are known to be involved in inflammatory responses, wound healing, and host defenses, the influence of these cells on choroidal inflammation is not well understood, and the physiological and pathological roles of choroidal mast cells remain unclear. Accordingly, we investigated the effects of various mast cell mediators on retinal pigment epithelial (RPE) cells in vitro. We hypothesized that mast cells might influence RPE cells via secreted mediators rather than cell contact-dependent mechanisms, because only a few mast cells are observed around the choroidal capillaries near Bruch's membrane despite the high number of these cells in the choroid. Therefore, we designed in vitro studies to evaluate interactions between RPE cells and mast cells via secreted mediators. First, we used the reverse transcription polymerase chain reaction (RT-PCR) to examine RPE cell expression of receptors for mediators produced by mast cells, such as tryptase, histamine, TNF-*α*, and various chemokines. Next, we assessed the effect of mast cell mediators, particularly tryptase, on the production of interleukin 8 (IL-8) and vascular endothelial growth factor (VEGF) by RPE cells, by using an antibody array and enzyme-linked immunosorbent assay (ELISA). We also investigated the effect of tryptase on phosphorylation of extracellular signal-regulated kinases (ERK) 1/2 by using an antibody array. Furthermore, we determined the influence of tryptase on RPE cell migration in the scratch assay. Finally, we examined the effect of tryptase on epithelial integrity and expression of tight junction-related proteins by RPE cells by measurement of transepithelial resistance (TER) and immunocytochemistry. To the best of our knowledge, this is the first report about the effects of mast cell mediators, particularly tryptase, on RPE cells.

## 2. Materials and Methods

### 2.1. Preparation of RNA and RT-PCR

We examined the expression of protease-activated receptor 2 (PAR2), histamine receptor 1 (HR1), and TNF-*α* receptor 1 (TNF-*α*R1) by employing appropriate primers for RT-PCR (Table 1). Total RNA was isolated from RPE cells using NucleoSpin® RNA II (Takara Bio Inc., Shiga, Japan). Then reverse transcription was carried out with ReverTra Ace-α-® (Toyobo Co., Ltd., Osaka, Japan), using approximately 1.5 *μ*g of total RNA in a final volume of 20 *μ*l. Subsequently, amplification was done with a Qiagen® Multiplex PCR Kit (Qiagen, Hilden, Germany) and 10 *μ*M of each primer for PAR2, HR1, and TNF-*α*R1 in a final volume of 20 *μ*l. PCR involved initial denaturation at 95°C for 15 min, followed by 35 cycles of denaturation at 94°C for 45 s, annealing at 60°C for 90 s, and polymerization at 72°C for 60 s, with final extension at 72°C for 10 min. Then, 3 *μ*l of each PCR product was subjected to 1.5% agarose gel electrophoresis and the gel was stained with ethidium bromide (0.4 ng/ml).

We also examined the expression of various chemokine receptors by RPE cells using multiplex PCR kits (Human chemokine receptor CCR kit-1 and kit-2, Maxin Biotech Inc., San Francisco, CA, USA) designed to detect the expression of human CCR1, CCR2, CCR3, CCR4, CCR5, CCR8, V28, CCR11, CCR6, CCR7, and GAPDH genes by identifying PCR products of 500 bp (GAPDH), 363 bp (CCR1), 318 bp (CCR3), 287 bp (CCR4), 246 bp (CCR5), 163 bp (CCR2), 428 bp (CCR8), 349 bp (V28), 302 bp (CCR11), 259 bp (CCR6), and 214 bp (CCR7).

### 2.2. Cell Culture

A human RPE cell line (ARPE 19) was purchased from the American Type Culture Collection (Manassas, VA) and cells were cultured in Dulbecco's modified Eagle's medium (DMEM) with 10% fetal bovine serum (FBS).

### 2.3. Transepithelial Resistance Assay

The integrity of RPE cell monolayers was determined by measuring transepithelial resistance (TER) with an EVOM voltmeter (EMD Millipore Corporation Billerica, MA). RPE cells were incubated in a 55 cm^2^ tissue culture dish until confluent, seeded at 1 × 10^5^ cells/ml on Transwell® polyester filters (0.4 *μ*m pore), and cultured until a polarized monolayer formed with a constant TER (incubation from 1 to 20 days at 37°C in a humidified atmosphere with 5% CO_2_ and medium supplementation on alternate days). Polarized RPE cell monolayers were incubated in a serum-free medium for 24 hours. Then tryptase (0.22 or 2.2 nM) was added to the apical compartment of the Transwells and incubation was done for 24 h, with measurement of TER after 1, 2, 3, 4, and 24 h. We chose these concentrations of tryptase according articles of TER incubated with tryptase [6, 7]. Results were calculated as the percent change relative to untreated control cells.

### 2.4. Antibody Array Analysis

Culture supernatant of RPE cells was analyzed with an antibody array (Ray Bio; Human Inflammation Antibody III kit; Ray Biotech Inc., Norcross, CA) according to the manufacturer's instructions. RPE cells were grown to subconfluence in a medium, washed twice with phosphate-buffered saline (PBS), and incubated in a serum-free medium for 24 hours with or without tryptase (10 nM). Then the culture supernatant was harvested for antibody array analysis. We also investigated the phosphorylation of mitogen-activated protein kinases (MAPKs) by using the Human Phospho-MAPK Array Kit Proteome Profiler™ (R&D Systems, MN, USA). RPE cells were incubated in a serum-free medium with or without tryptase (10 nM) for 1 h, followed by harvesting and lysis for antibody array analysis according to the manufacturer's instructions.

### 2.5. In Vitro Scratch Assay

RPE cells were grown to confluence in 6-well plates. A uniform wound was made in the cell layer on each plate using a 200 *μ*l pipette tip, followed by washing with PBS, and the influence of tryptase (36 nM, or 180 nM) on cell migration was assessed by observation and photography at 24 hours after wound creation. To quantitatively evaluate wound healing, NIH image software was used to measure total cell migration.

### 2.6. Enzyme-Linked Immunosorbent Assay

RPE cells were grown to subconfluence and then incubated in serum-free medium for 24 h with or without tryptase, histamine, TNF-*α*, MIP-1*α*, eotaxin, or I-309 at various concentrations. Then, the supernatant was harvested to detect IL-8 and VEGF using an ELISA kit (Quantikine; R&D Systems, MN, USA) according to the manufacturer's instructions.

### 2.7. Immunocytochemistry

Rabbit polyclonal antibody for junctional adhesion molecule-A (JAM-A), mouse monoclonal antibody for zonula occludens-1 (ZO-1), Goat anti-rabbit Alexa 594 conjugated antibodies, and Goat anti-mouse Alexa 488 conjugated antibodies were purchased from Invitrogen (Carlsbad, CA). Polarized monolayers of RPE cells were grown in a 24-well dish containing one circle cover slip of glass (12 mm in diameter). Cells were fixed with cold methanol for 3 min, rinsed twice with prewarmed PBS, and blocked by 2% bovine serum albumin (BSA) in PBS for 60 min. Then, cells were incubated with the primary antibodies, rabbit anti JAM-A antibody (10 *μ*g/ml) and mouse anti-ZO-1 antibody (10 *μ*g/ml), for 1 h at room temperature, washed three times with 2% BSA in PBS, and incubated with secondary antibodies (1 : 50–1 : 100) for 1 h at room temperature.

### 2.8. Statistical Analysis

Results are presented as the mean ± SEM. Comparisons between two groups were performed with unpaired Student's *t*-test. All analyses were done with Stat View for Windows software (Version 5.0; SAS Institute, Cary, NC), and *p* < 0.05 was considered to indicate significance.

## 3. Results

### 3.1. Expression of RAR-2, HR1, and TNF-*α*R1 by RPE Cells

**Figure 1:**
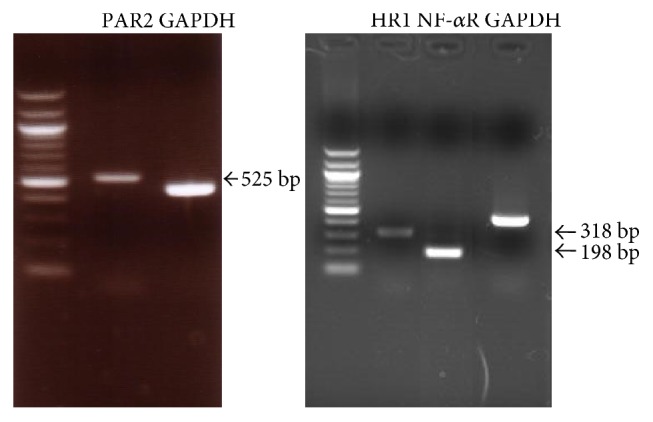
Expression of PAR2, HR1, and TNF-*α*R1 mRNA by RPE cells. PAR2, HR1, and TNF-*α*R1 products of the predicted sizes (525 bp, 318 bp, and 198 bp, resp.) were amplified from cellular RNA.

RT-PCR demonstrated the expression of PAR2, HR1, and TNF-*α*R1 mRNA by RPE cells (Figure 1).

### 3.2. CCR Expression

**Figure 2:**
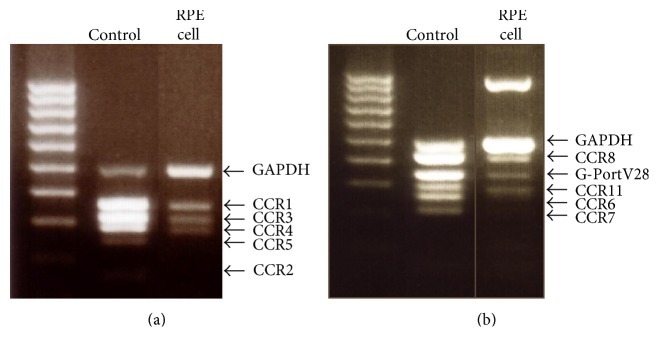
Expression of CCR mRNA by RPE cells. PCR products of the predicted sizes for CCR1, CCR3, CCR4, and GAPDH (363 bp, 318 bp, 287 bp, and 500 bp, resp.) were amplified from cellular RNA (a). PCR products of the predicted sizes for CCR8, V28, and CCR11 (428 bp, 349 bp, and 320 bp, resp.) were amplified from cellular RNA (b). No bands for CCR5, CCR2, CCR6, or CCR7 were detected.

We used multiplex PCR to investigate expression of CCRs by RPE cells. Gel electrophoresis revealed bands corresponding to the expected sizes of CCR1, CCR3, CCR4, CCR8, V28, and CCR11, while there were no bands for CCR2, CCR5, CCR6, or CCR7 (Figure 2).

### 3.3. Effect of Mast Cell Mediators on IL-8 Production by RPE Cells

**Figure 3:**
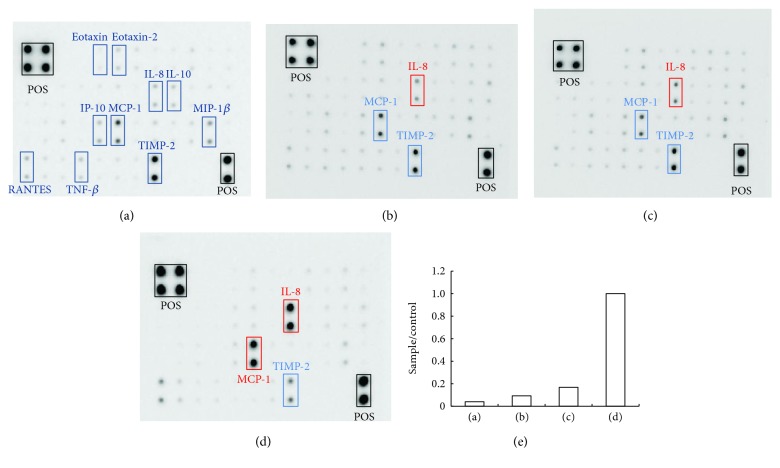
Antibody array analysis of culture supernatants from RPE cells stimulated by tryptase, histamine, or TNF-*α*. Representative arrays are shown. RPE cells were incubated in each medium for 24 h. (a) Incubation with only serum-free medium. (b) Incubation with serum-free medium containing tryptase (10 nM). (c) Incubation with serum-free medium containing histamine (200 *μ*g/ml). (d) Incubation with serum-free medium containing TNF-*α* (10 ng/ml). Cells constitutively produced IL-8, MCP-1, and TIMP-2. Incubation with tryptase, histamine, or TNF-*α* enhanced IL-8 production (red square) and TNF-*α* also enhanced MCP-1 production (red square). (e) The mean optical intensity of IL-8 positive spots was assessed.

**Figure 4:**
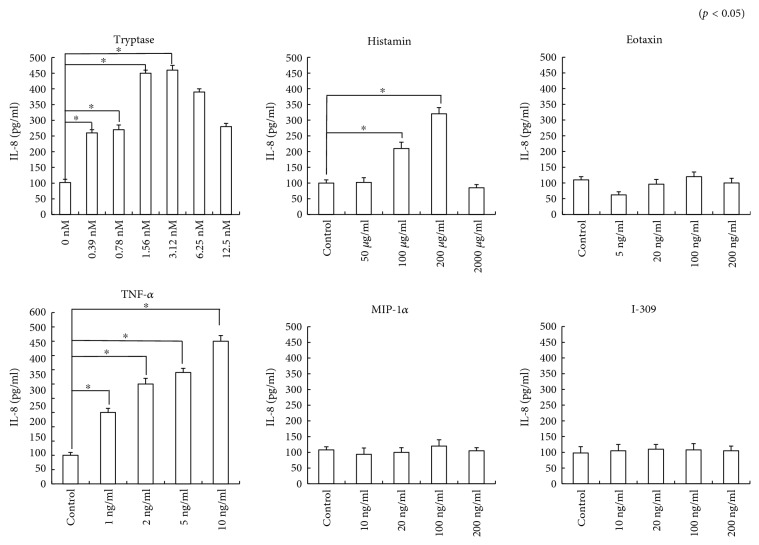
IL-8 production by RPE cells stimulated with mast cell mediators. ELISA showed constitutive IL-8 production by the cells. RPE cells were incubated with or without tryptase, histamine, TNF-*α*, eotaxin, MIP-1*α*, or I-309 at various concentrations for 24 h. IL-8 production was significantly enhanced by tryptase, histamine, or TNF-*α* in a concentration-dependent manner, while eotaxin, MIP-1*α*, and I-309 did not influence IL-8 production. Data are shown as the mean ± SEM of three independent experiments. ^∗^*p* < 0.05, significantly different from the control.

Culture supernatant was analyzed with an antibody array, and the mean optical intensity of positive spots was estimated, revealing constitutive production of IL-8, MCP-1, and TIMP-2 by RPE cells (Figure 3(a)). Treatment of the cells with tryptase (10 nM), histamine (200 *μ*g/ml), or TNF-*α* (10 ng/ml) enhanced the production of these substances (Figures 3(b), 3(c), and 3(d)). To examine the effects of mast cell mediators on IL-8 production, RPE cells were incubated with or without tryptase, histamine, TNF-*α*, eotaxin, MIP-1*α*, or I-309 at various concentrations for 24 h. It was found that tryptase, histamine, and TNF-*α* enhanced IL-8 production (Figure 4).

### 3.4. Effect of a PAR2 Agonist on IL-8 Production

**Figure 5:**
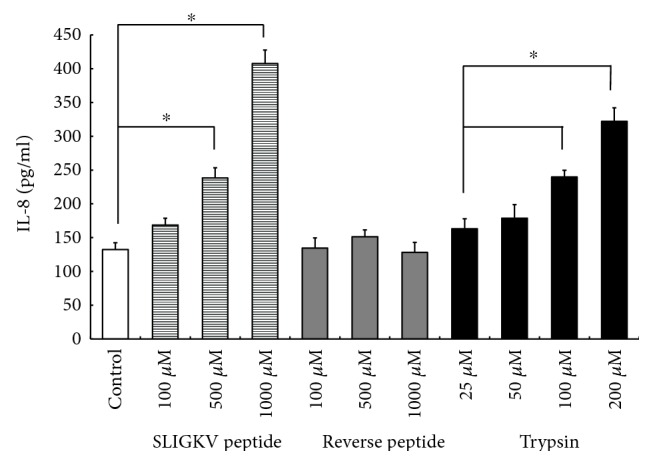
IL-8 production by RPE cells stimulated with PAR2 agonist. IL-8 production was examined after cells were stimulated with a PAR2 agonist peptide (SLIGKV), a decoy PAR2 agonist peptide (reverse peptide, LSIGKV), or trypsin. RPE cells were incubated in each medium for 24 h. Both the PAR2 agonist and trypsin enhanced IL-8 production in a concentration-dependent manner, but the decoy PAR2 agonist did not. Data are shown as the mean ± SEM of three independent experiments. ^∗^*p* < 0.05, significantly different from the control.

To confirm that the increase of IL-8 production by RPE cells treated with tryptase was dependent on PAR2, we examined IL-8 production when cells were incubated with or without a PAR2 agonist (SLIGKV), a decoy PAR2 agonist (reverse peptide, LSIGKV), or trypsin (which is also a ligand of PAR2). Both the PAR2 agonist peptide and trypsin enhanced IL-8 production in a concentration-dependent manner, while the decoy PAR2 agonist did not increase IL-8 production (Figure 5). These results suggested that tryptase acted via PAR2 to enhance the production of IL-8 by RPE cells.

### 3.5. Effect of Tryptase on VEGF Production

**Figure 6:**
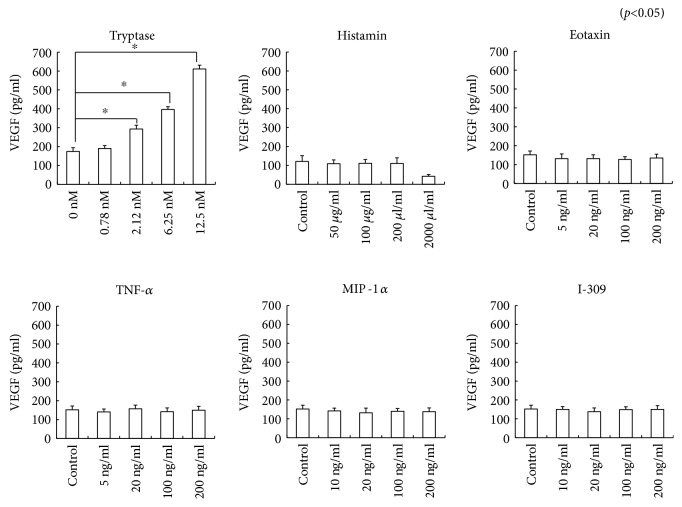
VEGF production by RPE cells simulated with mast cell mediators. ELISA revealed constitutive production of VEGF. RPE cells were incubated with or without tryptase, histamine, TNF-*α*, eotaxin, MIP-1*α*, or I-309 at various concentrations for 24 h. Tryptase enhanced VEGF production in a concentration-dependent manner, while the other mast cell mediators did not. Data are shown as the mean ± SEM of three independent experiments. ^∗^*p* < 0.05, significantly different from the control.

To examine the effects of various mast cell mediators on VEGF production, RPE cells were cultured with or without tryptase, histamine, TNF-*α*, eotaxin, MIP-1*α*, or I-309 at various concentrations for 24 h. It was found that only tryptase enhanced VEGF production by the cells in a concentration-dependent manner, while the other mediators did not increase VEGF production (Figure 6).

### 3.6. Phosphorylated ERK 1/2 Expression after Tryptase Stimulation

**Figure 7:**
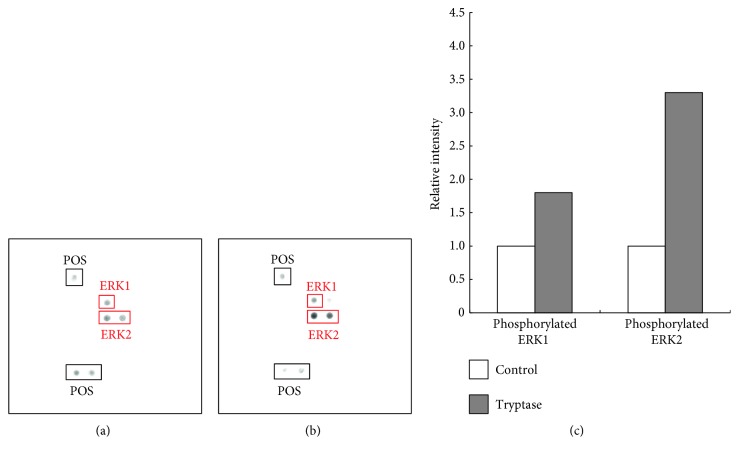
Expression of phosphorylated ERK 1/2 by tryptase-stimulated RPE cells. Cell lysates were analyzed with an antibody array and representative results are shown. (a) Incubation with serum-free medium. (b) Incubation with tryptase (10 nM) for 1 h. Cells constitutively expressed phosphorylated-ERK 1/2, and tryptase stimulation increased phosphorylated-ERK 1/2 expression (red square). The mean optical intensity of positive spots was estimated (c).

When lysates of RPE cells were analyzed with an antibody array, these cells were found to constitutively express phosphorylated-ERK 1/2. After stimulation with tryptase (10 nM), expression of phosphorylated-ERK 1/2 increased (Figure 7).

### 3.7. Effect of Tryptase on Cell Migration

**Figure 8:**
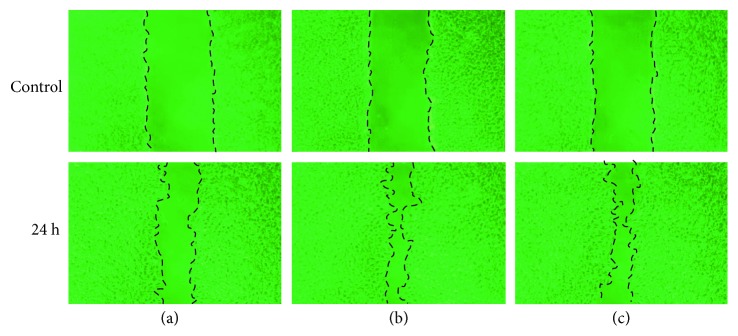
Cell migration in the scratch assay. The scratch assay assessed in vitro migration of RPE cells stimulated by tryptase (36 nM and 180 nM). A uniform wound was made in the cell monolayer in each plate using a 200 *μ*l pipette tip and was observed after 24 h. To quantitatively evaluate wound healing, total cell migration was determined with NIH image. (a) Control, (b) tryptase (36 nM), and (c) tryptase (180 nM). Tryptase induced RPE cell migration.

To examine the influence of tryptase on migration of RPE cells, the scratch assay was performed on subconfluent cultures of RPE cells in 6-well plates. It was demonstrated that incubation with tryptase (36 nM or 180 nM) for 24 h increased cell migration into the wound (Figure 8). We also demonstrated 10 nM of tryptase; it did not increased cell migration into the wound.

### 3.8. Effects of Tryptase on Epithelial Integrity and Expression of ZO-1 and JAM-A

**Figure 9:**
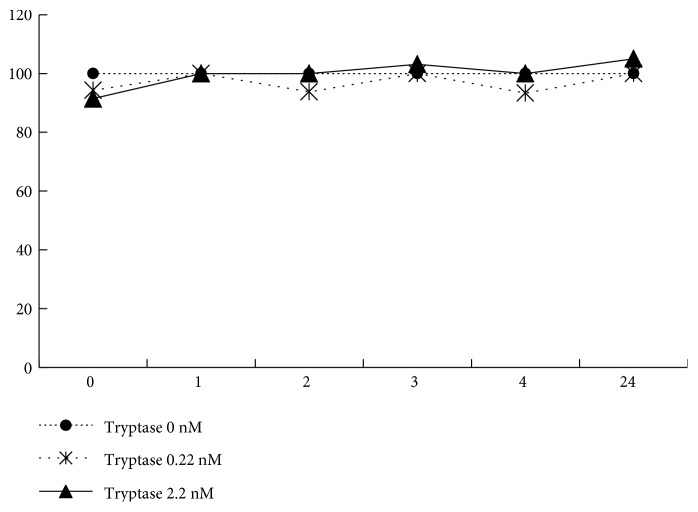
Effect of tryptase on the epithelial barrier. Transepithelial resistance (TER) of RPE cells for 24 h incubated with tryptase (0.22 or 2.2 nM) compared with that of untreated control cells. There was no change of TER.

**Figure 10:**
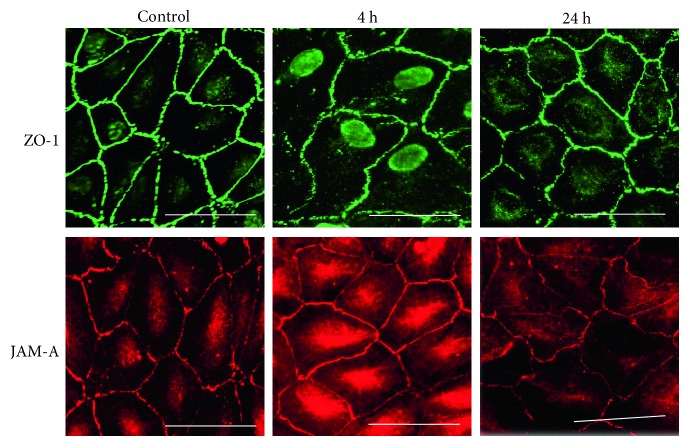
Expression of ZO-1 and JAM-A proteins. RPE cells were incubated with tryptase (0.22 or 2.2 nM) for 4 h or 24 h. The expression of ZO-1 and JAM-A was estimated by immunocytochemistry. There were no differences of the expression between RPE cells treated with tryptase and control. Bars = 20 *μ*m.

Incubation with tryptase for 24 h did not alter the epithelial integrity of the RPE cell monolayer because there was no change of TER, even at high-tryptase concentrations (Figure 9). Immunocytochemistry revealed that incubation with tryptase (2.2 nM) for 4 or 24 h did not alter the expression of ZO-1 or JAM-A by RPE cell monolayers, which corresponded to the findings for TER (Figure 10).

## 4. Discussion

We investigated the expression of PAR2, HR1, TNF-*α*R1, and various CCRs by RPE cells. Because the major secretory mediators stored in mast cell granules are tryptase, histamine, and TNF-*α*, it is possible that these mediators could influence RPE cells after mast cell degranulation in the choroid.

The main type of tryptase released by mast cells is *β*-tryptase. Tryptase is a serine protease that acts as a ligand for PAR2, which is activated when a serine protease binds to and cleaves the amino terminal domain of the receptor, thus generating a new N-terminus that functions as a tethered ligand and binds with the receptor to initiate transmembrane signaling. PAR2 can also be activated by specific PAR2 agonist peptides, such as SLIGKV, which mimic the tethered ligand. Inverting the first two amino acids of the tethered ligand from SLIGKV to LSIGKV results in loss of function of this peptide, confirming its high specificity for PAR2 [8]. In the present study, IL-8 production was induced by incubating RPE cells with tryptase, trypsin, or SLIGKV, but not LSIGKV. These results indicate that tryptase enhanced IL-8 production via PAR2. We next investigated the influence of tryptase on VEGF production by RPE cells. We found that tryptase induced VEGF production, while histamine, TNF-*α*, and other chemokines did not. These results suggest that tryptase produced by choroidal mast cells may have an important role in the processes of inflammation and angiogenesis in the RPE layer. Furthermore, we showed that treatment with tryptase increased the migration of RPE cells. There have been several reports that RPE cell migration is dependent on phosphorylation of ERK 1/2 [9, 10]. Phospho-ERK 1/2 is considered to be a surrogate marker of PAR2 activation and is associated with tryptase-induced activation of PAR2 in fibroblasts or colonic epithelial cells [11–13]. In the present study, tryptase induced the rapid phosphorylation and activation of ERK 1/2 in RPE cells, indicating that functional PAR2 receptors are expressed by RPE cells and can be stimulated by tryptase, leading to activation of ERK 1/2 and increased cell migration.

It is well known that TNF-*α* plays a primary role in uveitis. TNF-*α* levels are high in the aqueous humor and serum of patients with Behcet's disease, as well as in the aqueous humor and serum of rats with endotoxin-induced uveitis (EIU) [14]. Moreover, intravitreal injection of TNF-*α* induces acute uveitis in mice and rats associated with infiltration of polymorphonuclear cells into the uveal tissue [15, 16]. Furthermore, Silva et al. reported that mast cells are a potential source of pharmacological mediators strongly related to the pathophysiology of EIU in rats [4]. In this study, we demonstrated the expression of TNF-*α*R1 by RPE cells and showed that TNF-*α* strongly enhances RPE cell expression of IL-8. Therefore, it is possible that TNF-*α* produced by choroidal mast cells and infiltrating macrophages has an important role in the pathogenesis of uveitis.

The pathophysiological effects of histamine are mediated through four distinct G-protein-coupled receptors (H_1_R, H_2_R, H_3_R, and H_4_R). Among them, H_3_R and H_4_R are predominantly expressed in the central nervous system and hematopoietic cells, respectively, whereas H_1_R and H_2_R are expressed ubiquitously [17, 18]. We found that H_1_R was expressed by RPE cells, suggesting that mast cell degranulation in the choroid could have a strong impact on RPE cells via histamine release, but the role of histamine in this setting is not well known. Zawinka et al. reported that intravenous administration of histamine increases blood flow in the choroid [19–21], suggesting that mast cells may regulate choroidal blood flow. In the present study, we demonstrated that histamine enhances IL-8 production by RPE cells, raising the possibility that histamine released from mast cells may contribute to RPE inflammation.

We also demonstrated that RPE cells express CCR1, CCR3, CCR4, CCR8, and CCR11, while CCR2, CCR5, CCR6, and CCR7 were not expressed by these cells. Nakajima et al. examined the expression of more than 1000 genes by cultured human mast cells using a high-density oligonucleotide probe array [22]. They found that activation of high-affinity IgE receptor 1 (Fc*ε*R1) markedly increased the transcription of several CC chemokines, with CCL1 (I-309), CCL3 (MIP-1*α*), and CCL4 (MIP-1*β*) being among the 10 most strongly upregulated transcripts from approximately 12,000 genes [22]. Another CC chemokine, CCL2 (MCP-1), is strongly expressed by cultured human mast cells in the resting state [22]. CCL1 recruits Th2 cells and its receptor is CCR8, while CCL3 recruits Th1 cells and its receptor is CCR1. CCR3 is also the receptor for CCL11 (eotaxin). In this study, we demonstrated that cultured RPE cells expressed CCR8, CCR1, and CCR3. However, we also found that CCL1, CCL3, and CCL11 did not enhance the production of IL-8 or VEGF by RPE cells. Therefore, CC chemokines produced by mast cells may not modulate RPE cells during inflammation and angiogenesis.

Finally, we investigated the effect of tryptase on epithelial integrity and on the expression of JAM-A and ZO-1 by RPE cells.

The influence of tryptase on epithelial integrity has been controversial. Jacob et al. reported that tryptase regulates the integrity of intestinal epithelium [23], but Chang et al. found that tryptase did not influence the integrity of human airway epithelium [6]. Wilcz-Villega et al. demonstrated that tryptase disrupted the integrity of intestinal epithelial cells monolayers, causing a significant decrease of TER and a decrease of JAM-A and ZO-1 expression within 24 h, while inhibition of tryptase ameliorated the decrease of JAM-A expression and TER [7]. JAM-A is expressed by several types of cells, and it functions as a gatekeeper that regulates permeability across both endothelial and epithelial monolayers, as well as leukocyte transmigration through endothelium. Therefore, Wilez-Villega et al. concluded that reduction of JAM-A expression may contribute to impairment of epithelial integrity by tryptase. In contrast, we found that tryptase did not alter epithelial integrity or JAM-A expression by RPE cells. These discrepancies between the two studies may be related to investigation of different types of cells, suggesting that we should study primary RPE cells in the future. In conclusion, the present in vitro investigation provided evidence that tryptase and other mediators released from mast cells may contribute to inflammation and angiogenesis in the RPE. Further investigation of choroidal mast cell function may lead to new prospects for therapeutic intervention.

## Figures and Tables

**Table 1:** List of the primers used in this study.

Gene	Primer	Primer sequence	Product length (bp)
PAR2	Forward	5′-CCCTTTGTATGTCGTGAAGC-3′	525
	Reverse	5′-TTCCTGGAGTGTTTCTTTGAGG-3′	
H R 1	Forward	5′-GACTGTGTAGCCGTCAACCGGA-3′	318
	Reverse	5′-TGAAGGCTGCCATGATAAAACC-3′	
TNFR1	Forward	5′-ACCGGCATTATTGGAGTGAAAA-3′	198
	Reverse	5′-GGGGTAGGCACAACTTCGTG-3′	
